# Comparing the Driving Skills of Adolescents with Obstructive Sleep Apnea to Healthy Controls: The Results of a Case-Controlled Observational Study

**DOI:** 10.3390/children10101624

**Published:** 2023-09-29

**Authors:** Andrea L. Fidler, Nanhua Zhang, Narong Simakajornboon, Jeffery N. Epstein, Shelley Kirk, Dean W. Beebe

**Affiliations:** 1 Division of Behavioral Medicine and Clinical Psychology, Cincinnati Children’s Hospital Medical Center, Cincinnati, OH 45229, USA; 2 Division of Biostatistics and Epidemiology, Cincinnati Children’s Hospital Medical Center, Cincinnati, OH 45229, USA; 3 Department of Pediatrics, University of Cincinnati College of Medicine, Cincinnati, OH 45229, USA; 4 Sleep Center, Division of Pulmonary Medicine, Cincinnati Children’s Hospital Medical Center, Cincinnati, OH 45229, USA; 5 The Center for Better Health and Nutrition of the Heart Institute, Cincinnati Children’s Hospital Medical Center, Cincinnati, OH 45229, USA

**Keywords:** obstructive sleep apnea, driving risk, sleepiness, inattention, adolescents

## Abstract

Auto crashes are a leading cause of death and injury among adolescents. Untreated obstructive sleep apnea (OSA) can cause sleepiness and inattention, which could negatively impact novice drivers, but OSA-related studies have focused on older drivers. This study used a driving simulator to examine whether licensed 16–19-year-old adolescents with OSA have diminished driving skills. Twenty-one adolescents with OSA and twenty-eight without OSA (both confirmed using polysomnography) completed two randomly ordered driving trials in a simulator (with induced distractions versus without). A mixed ANOVA examined the between-subjects effect of the OSA group, the within-subjects effect of the distraction condition, and the group-by-condition interaction effect on the ability to maintain lane position and the frequency of extended eye glances away from the roadway. T-tests were also used to examine group differences in reported sleepiness and inattention during daily life. The distraction task increased extended off-road glances and difficulties maintaining lane position (*p* < 0.001). However, adolescents with OSA did not display worse eye glance or lane position than controls and there were no significant group-by-condition interactions. Although the groups differed on polysomonographic features, there were also no significant differences in reported sleepiness or inattention. The distraction task negatively impacted both groups of adolescent drivers, but those with OSA did not fare differentially worse. Most adolescents in our study had mild OSA (median obstructive apnea–hypopnea index = 4.4), the most common form in the community. It remains possible that youth with more severe OSA would show increased driving impairment.

## 1. Introduction

Automobile crashes are the leading cause of death among teenagers and are a major cause of non-fatal injuries [1,2,3]. In addition to the emotional and physical toll, adolescent automobile crashes have a substantial financial impact, costing the US society approximately $11 billion per year [3]. The crash rate for adolescent drivers is higher than at any other age [1]. This has led to major public health efforts including intensive education, improved vehicle and roadway design, graduated licensing, restrictions on late-night driving, and bans on high-risk behaviors (e.g., driving while texting or after drinking alcohol). Despite these efforts, the crash-related adolescent death rate in the US has continued to rise [1]. Given the high cost of adolescent crashes, there is a clear need to identify risk factors that are treatable but often go unaddressed.

Obstructive sleep apnea (OSA) is a condition in which the upper airway repeatedly collapses or is blocked during sleep, resulting in poor sleep quality, hypoxia, and hypercarbia [4]. Risk factors for pediatric OSA include obesity, adenotonsillar hypertrophy, inflammation, craniofacial anomalies (e.g., malocclusions), and neuromuscular disorders [5,6,7]. If left untreated, OSA can lead to long-term cardiovascular and metabolic consequences [8,9]. However, it is the immediate effects of OSA on daytime sleepiness and inattention that contribute to the 2–3-fold increase in crash risk for adults with OSA [10,11,12,13,14]. Among adults, OSA has been estimated to contribute to 800,000 car crashes in the US each year [15]. The most effective countermeasure is OSA treatment, which has been shown to normalize driving in adults [11,14].

Although over 2 million teenagers in the US may have OSA [16,17,18,19], adolescent OSA is often overlooked. In one study that showed diminished attention in daily life among adolescents with OSA, over 80% of the OSA sample had never previously undergone formal sleep evaluation [20]. There are no published estimates of the number of crashes that are caused by OSA in adolescents, but there are multiple reasons to believe they might occur. Crash rates are highest among teens who report inadequate sleep duration or poor sleep quality [21,22]. Adolescents who self-report OSA symptoms, such as loud snoring, also report elevated rates of nodding off while driving [23]. Additionally, experimentally restricting sleep worsens teens’ driving skills, in part due to inattention [24], and adolescents with OSA experience inattention that improves with positive airway pressure (PAP) treatment [20,25]. Taken together, these findings suggest that OSA may be an unaddressed contributor to adolescent crashes. 

Prior studies examining the impact of OSA on driving have focused on middle-aged drivers with decades of experience [11]. We cannot assume these findings are equally applicable to adolescents, for several reasons. Adolescents show neurobehavioral deficits at levels of OSA severity that are subthreshold by adult criteria [20]. Even more importantly, the act of driving differs for adolescents and adults. Driving is a complex task that requires the continuous updating of roadway conditions, awareness of the position of dynamic obstacles in space, the interpretation of changing roadway signs and lane markers, frequent dashboard and mirror checks, and the ongoing adjustment of automobile controls (e.g., steering and pedals) [26,27,28]. Experienced adult drivers are so automatic in these skills that they often execute them unconsciously. Novice drivers lack that automaticity, so the demands on their attention are much greater [26,27,28]. Also, experienced drivers rarely exhibit extended glances away from the roadway, but novice teen drivers frequently do so [29,30,31,32]. This makes adolescents more vulnerable to distractions [27,33,34]. It may also increase their vulnerability to conditions such as OSA that degrade alertness and attention [20,35,36]. However, the effect of OSA on adolescent driving has not yet been tested.

This case-controlled observational study explored the impact of OSA on the driving skills of adolescents. To maximize application to the real world, licensed 16–19-year-old drivers were recruited primarily from the general community (rather than a clinical sample), then assessed for OSA using formal polysomnography (PSG). Those with versus without OSA were compared on performance in a driving simulator, allowing for precise measurements and ethically presented driving challenges, including secondary distraction tasks. In doing so, this study aimed to test whether (1) adolescent OSA increases driving risk, and (2) inattention and daytime sleepiness are mechanisms in OSA-linked driving risk. We hypothesized that, compared to demographically similar healthy controls, (1) adolescents with OSA are less consistent in maintaining lane position, especially during periods of distraction, and (2) inattention and sleepiness statistically mediate the group effect on simulated driving.

## 2. Materials and Methods

### 2.1. Participants and Group Determination

Fully licensed automobile drivers aged 16–19 years old were recruited primarily via social media advertising, supplemented by ads across a regional hospital network. Adolescents were excluded if they (1) had a diagnosed neurological illness, craniofacial condition affecting the airway, intellectual disability, autism, bipolar disorder, or psychosis; (2) took any medications known to substantially affect sleep (e.g., stimulants); (3) had undergone surgical or other treatment for OSA within the past two years; or (4) were non-fluent in English. 

As part of a pre-screening process, caregivers completed the PSQ [36] and reported the adolescent’s sex, height, and weight. We used the published threshold of 0.33 on the sleep-related breathing disorder subscale of the PSQ to identify those at low vs. high risk for OSA. That threshold was previously established using receiver–operator curve calculations to determine the optimal cutoff to maximize test sensitivity and specificity to OSA [37]. The PSQ has demonstrated a high ability to predict PSG-defined obstructive sleep-related breathing disorders [37]. Parent-reported height and weight were used to calculate a preliminary body mass index (BMI). These pre-screening data were used to prioritize follow-up recruitment calls; the goal was to recruit OSA and control samples that were similar in sex and overall adiposity, realizing that one-to-one matching was impossible because self-report only roughly estimates the more rigorous objective metrics we used for the final group classification and calculation of BMI. 

The final group classification (OSA vs. control) was determined after each participant underwent formal hospital-based overnight PSG. PSG was performed in accordance with the American Academy of Sleep Medicine (AASM) guidelines in an AASM-accredited sleep laboratory at Cincinnati Children’s Hospital Medical Center. A standard pediatric montage was used with BWAnalysis (Neurovirtual, Fort Lauderdale, FL, USA). The following variables were recorded simultaneously: body position, left and right electrooculogram (ROC/A1, LOC/A2), 6-channel electroencephalogram (F4-M1, C4-M1, O2-M1, F3-M2, C3-M2, and O1-M2), chin electromyogram, electrocardiogram, pulse oximetry and pulse waveform (Masimo, Irvine, CA, USA), thoracic and abdominal inductance plethysmography (SleepSense, Elgin, IL, USA), airflow with thermistor (Salter Labs, El Paso, TX, USA) and nasal pressure transducer (Pro-Tech—Philips Respironics, Murrysville, PA, USA), and end-tidal pCO2 (BCI Capnocheck, Smiths Medical, St. Paul, MN, USA).

The PSG was scored by a board-certified sleep medicine physician who was blinded to other study measures to minimize the potential for bias. The AASM guidelines were used to score sleep staging, respiratory events, and arousals [38]. For group classification and descriptive purposes, we recorded the following PSG outcomes: obstructive apnea–hypopnea index (obstructive AHI), periodic limb movement index, arousal index, sleep efficiency after sleep onset, sleep period time (onset to offset), average oxygen saturation during NREM and REM, and percent of time in N1, N2, N3, and REM. Participants with an obstructive AHI ≥ 1.5 per hour were classified as meeting OSA diagnostic criteria as per the International Classification of Sleep Disorders (ICSD-3) [4], focusing on obstructive events and hypoventilation as defined by the pediatric criteria. Pediatric criteria were applied instead of adult criteria (which require more frequent events) because prior work suggests that adolescents meeting pediatric criteria show reduced attention in daily life [20]. The control group consisted of participants who were found not to have OSA using those criteria and who also fell below the PSQ threshold. Those who fell above the PSQ threshold but did not meet the clinical criteria for OSA were considered “simple snorers” and dropped from the analyses to promote a clearer comparison between the OSA and control groups [20].

### 2.2. Procedures

Adolescents and their caregivers arrived at an outpatient research space between 3 and 4 pm (the time of day when the most adolescent crashes occur [33]) for a 1.5 to 2 h visit in which they completed non-PSG study measures. Most visits occurred the afternoon immediately prior to PSG. Out of 49 total participants, 2 adolescents completed the evaluation and PSG on separate days within 1 month of one another. For adolescents who were recently diagnosed with OSA (*n* = 3), we obtained their PSG results and diagnoses from their medical record. Data collection occurred between September 2019 and March 2022. Adolescents and caregivers provided assent and informed consent, respectively. All procedures were approved by the hospital institutional review board.

**The driving simulation** was conducted on an STISIM Model 400 simulator equipped with a high-definition video monitor displaying the roadway (Systems Technology Incorporated, Hawthorne, CA, USA). This driving simulator has three driving displays to provide a 135-degree driver field-of-view with integrated rear-view and side mirrors, a full-sized steering wheel with dynamics-based feedback, full-sized foot pedals, and a fixed-base, adjustable full-sized car seat. Participants received real-time audio, visual, and haptic feedback on steering, braking, and acceleration. Adolescents’ eye gaze was tracked using the Tobii mobile eye tracking system (Tobii, Stockholm, Sweden). The Tobii eye tracker comprises a lightweight glasses frame outfitted with infrared reflective lenses that allow the position of the pupil to be tracked via retinal reflection. A forward-facing scene camera is fixed to the frame to detect the participant’s field of view. The position of the pupil is then registered with respect to the scene camera. This allows for the precise, real-time recording of the driver’s gaze vis à vis their environment. This system allowed us to safely present driving challenges in a relatively immersive environment while precisely recording the driver’s performance and eye gaze.

Participants completed two randomly ordered driving trials in a simulator—one with distractions and one without distractions. All participants began with a 15 min introduction to the simulator, including eye tracker setup and practice with our distraction task, explanation of the screens and controls, and a 5 min training drive to familiarize them with the “feel” of the simulator. Those who reported symptoms of “simulator sickness” (e.g., nausea) on the Georgia Tech Simulator Sickness Screening survey [39] after the 5 min training drive discontinued the study to avoid sustained discomfort. Those who remained in the study then completed two 15 min simulated drives that presented nearly identical driving scenarios. Both comprised a 2-lane roadway with modest curves and light traffic. Sidewalks with pedestrians, periodic groups of buildings, trees, and street signs lined the simulated roadway. Oncoming and crossing traffic did not enter the lane. This allowed for continuous driving without any starts, stops, cross-traffic, or unexpected events. Similar drives have been found to be sensitive to OSA in adults [40,41] and sleep restriction in adolescents [24]. 

The distraction task involved a street name search task using procedures outlined in our prior work [42]. On 14 semi-randomly timed occasions during each drive, adolescents heard a beep and a letter was displayed in the bottom right portion of the center screen. During the non-distraction drive, participants were instructed to simply state the letter aloud. During the distraction drive, they were instructed to count the number of streets on a map placed at the center console location that started with that letter. We calculated their accuracy on this secondary task as the number of mistakes on the street name search task. The goal was to simulate a task that demands attention away from the roadway, somewhat analogous to adjusting settings on a touch screen or reading a text message.

### 2.3. Measures

#### 2.3.1. Driving Safety (Aim 1)

Standard Deviation (SD) of Lane Position: Our primary driving safety outcome was the standard deviation of the distance from the simulated car to the center of the lane, which measures the participants’ ability to maintain lane position. This index of lateral vehicle control is commonly derived from driving simulators and is highly correlated with leaving one’s lane of traffic and the risk of an on-road accident [43,44]. It has also been shown to be sensitive to both adult and adolescent sleepiness [24,45,46,47]. Higher values indicate worse vehicle control. 

Self-Reported Driving History: As secondary outcomes, we examined the adolescents’ self-report of motor vehicle crashes and citations (e.g., speeding tickets) on the Barkley Driving Questionnaire [48]. These outcomes were secondary because they were based on self-report due to limitations in accessing official records for drivers under the age of 18. Outcomes were analyzed as ever having had a motor vehicle accident (yes vs. no) and ever having had a citation (yes vs. no).

#### 2.3.2. Inattention and Sleepiness (Aim 2)

Protracted Eye Glances Away from the Road: Our primary measure of driving-relevant inattention was the number of eye-tracker-defined glances away from the roadway that lasted >2 s. The 2 s threshold was chosen because such glances lead to 3.6 times more lane departures and have been implicated in the large majority of crashes [49,50]. Secondarily, we also examined the mean duration of those long glances from the roadway. To determine whether the adolescents’ visual glances were on or off the roadway for each 20 msec epoch, gaze analysis software (Tobii Pro Lab; Version 1.98.1) used eye tracking coordinates and an a priori researcher-defined forward roadway gaze area.

Inattention in Daily Life was primarily measured via the parent report on the Behavioral Assessment System for Children (BASC-3, parent report), a standardized questionnaire in which parents report on functioning over the past 6 months [51]. The parent-reported attention problems subscale of the BASC-3 has good test–retest reliability (*r* = 0.85), inter-rater reliability (*r* = 0.70), and internal consistency (α = 0.88) [51]. The inattention subscale of an earlier version of the BASC has been found to be sensitive to adolescent OSA [20]. Secondarily, we also examined parent and participant self-report on a Behavior Rating Form (BRF) that asked about 9 inattention symptoms over the past several days; the BRF has been shown to be sensitive to short-term sleep manipulation among adolescents [52,53]. The inattention subscale of the BRF correlates significantly with the Vanderbilt Assessment Scale, demonstrates strong internal consistency (α = 0.8–0.9), and demonstrates moderate stability across sessions with experimentally varied sleep schedules (rho = 0.24–0.67) [52,53]. In each case, raw scores were used to maximize meaningful variability within our narrow age range; higher scores indicate greater inattention.

Sleepiness was primarily measured via self-report on the Epworth Sleepiness Scale for Children and Adolescents (ESS-CHAD) [54]. The ESS-CHAD is a unidimensional scale that has demonstrated high internal consistency (α = 0.73) and test–retest reliability (r = 0.89) [54]. In adults, ESS scores correlate with nodding off at the wheel [55,56,57]. Secondarily, we also examined the 5-item sleepiness subscales from the parent- and self-reported BRF, which have been shown to be sensitive to experimental sleep manipulations [52,53]. The sleepiness subscale of the BRF has high levels of correlation with the Child Sleep Habits Questionnaire [52]. It has also demonstrated strong evidence of internal consistency (α ≥ 0.8) and moderate stability across experimental sleep conditions (rho = 0.24–0.67) [53]. Raw scores were again used, with higher scores indicating higher levels of sleepiness.

#### 2.3.3. Descriptive Measures and Potential Covariates

Parents provided demographic information, including the adolescents’ race, ethnicity, gender, academic grades, and family income. Adolescents reported their typical bed and wake times on school nights and weekends, allowing us to calculate time in bed and sleep midpoint on both school nights and weekends. Driving experience was calculated as the difference between the date of the driving simulation and the date an adolescent was issued a license to drive independently. At the study visit, study staff measured the adolescents’ height and weight using a calibrated hospital scale; these data were used to calculate BMI and the following BMI categories based on age and sex matched the Center for Disease Control growth charts: healthy (<85th percentile), overweight (85th to 94.9th percentile), obesity (95th percentile to 119.9% of 95th percentile), and severe obesity (>120% of 95th percentile).

### 2.4. Analyses

To test whether any descriptive variables might be a potential confounder, preliminary *t*-tests (continuous variables), Mann–Whitney U tests (non-normally distributed variables), and Chi-square tests (discrete variables) were used to compare participant characteristics between the OSA and control groups. We inserted driving experience and demographic variables as covariates into the primary analyses and retained them in the model if significant. For the Aim 1 primary analysis, we used repeated-measures analysis of variance (RM-ANOVA) to examine the between-subjects effect of the OSA group, the within-subjects effect of the distraction condition, and the group-by-condition interaction effect on the ability to maintain lane position. To control for any potential ordering effects, we included a variable accounting for the ordering of the distraction task. Aim 1 secondary analyses were conducted via *t*-tests or Mann–Whitney U tests. Because statistical mediation requires associations between the purported mediator and study group, Aim 2 analyses began by (a) repeating the RM-ANOVAs, with the dependent variables of frequency of protracted eye glances away from the roadway and the mean length of those protracted eye glances, and (b) comparing reported inattention in daily life and reported sleepiness across groups via *t*-tests. If group effects for Aims 1 and 2 were found to be significant, we planned to examine the mediating effects of inattention and daytime sleepiness using the method proposed by Preacher and Hayes for multiple mediators [58]. The RM-ANOVA models retained subjects who were missing some outcome data (detailed below) and provided correct inferences under the missing-at-random mechanism [59]. Statistical analyses were conducted in SAS version 9.4 (SAS Institute, Cary, NC, USA) and SPSS version 27 (IBM Corp, Armonk, NY, USA).

### 2.5. Sample Size and Power Analysis

The sample size target was based on detecting a significant difference in the standard deviation of distance from the simulated car to the center of the lane (SD of the lane position) between OSA and control groups. At the time the study was designed, studies in adults with OSA had indicated large effects of OSA on the SD of lane position (Cohen’s d = 0.67 to 1.81, all but one d > 1.0) [45,46,47,60,61]. Assuming an effect size of 0.8, the target sample size of 50 participants achieves > 80% power to detect a group difference using a two-sided two-sample *t*-test.

## 3. Results

### 3.1. Participants and Preliminary Analyses

**Figure 1:**
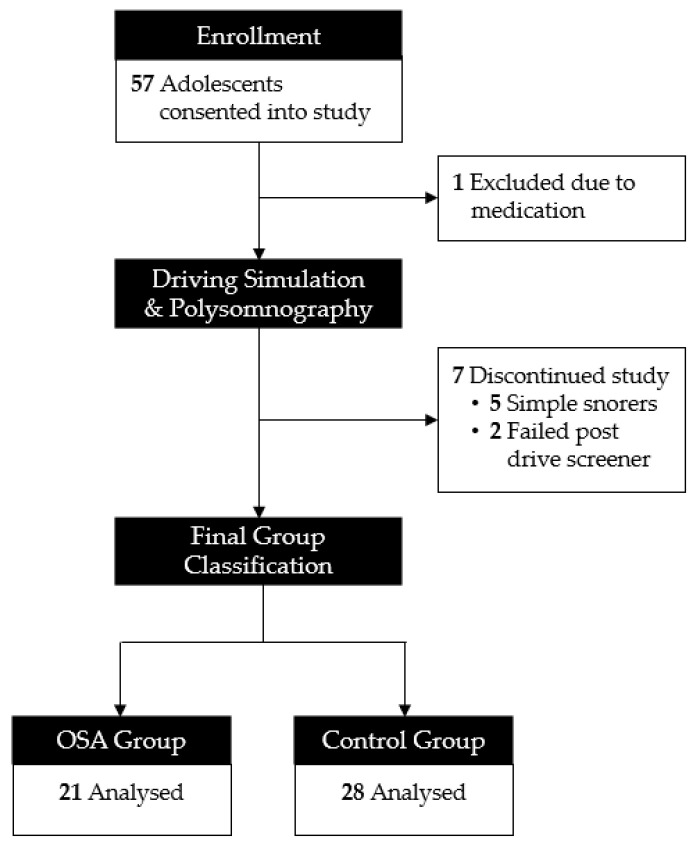
Consort diagram.

Fifty-seven adolescents consented to participate in the study. One was excluded due to the use of stimulant medication. Seven adolescents were discontinued due to being simple snorers (defined above; *n* = 5) or due to reporting symptoms of “simulator sickness” following the pre-drive (*n* = 2). Our final sample included 21 adolescents with OSA and 28 controls without OSA (see Figure 1). 

Table 1 displays each group’s demographic features, PSG data, and self-reported sleep patterns. Most adolescents (M_age_ = 17.68 ± 0.92; range = 16–19 years) were White (89.8%), lived with both biological parents in the same home (67.3%), and had a household income > $100K (63.2%). Despite initial efforts to match on self-reported adiposity, the OSA group had more adolescents with higher BMIs than the control group, *p* = 0.02; this is consistent with prior research showing that adolescents with obesity have higher rates of OSA [19]. There were no other statistically significant differences between the OSA group and controls on any other demographic variables. As expected, adolescents with OSA had a higher obstructive AHI (median = 4.40; range = 1.5–24.9) than controls (median = 0.45; range = 0.00–1.30), *p* < 0.001. Adolescents with OSA also had a higher arousal index (median = 12.3; range = 3.5–21.5) than controls (median = 6.30; range = 2.7–12.4), *p* < 0.001. When compared to controls, the OSA group spent less time in Stage N3 (slow wave sleep; *p* = 0.004) and more time in Stage N1 (light/transitional sleep; *p* = 0.035).

### 3.2. Aim 1: Impact of OSA on Driving

In primary analyses, there was no significant difference in ability to maintain lane position between those with OSA and the controls, *F*(1, 43) = 0.83, *p* = 0.37 (see Table 2). In contrast, the effect of the distraction task was strikingly significant, *F*(1,43) = 54.64, *p* < 0.001, such that adolescents had more difficulty maintaining lane position during the distraction drive. The group-by-condition interaction trended towards significance, *p* = 0.06. Exploratory post hoc comparisons revealed that, while both groups were affected by the distraction task, the effect was somewhat smaller in the OSA group (mean standardized change = 0.23; *p* < 0.001) than the control group (mean standardized change = 0.38; *p* < 0.0001). Notably, we ran the primary analyses including teen age, gender, race/ethnicity, BMI, driving experience, and family income as covariates; none of the covariates were statistically significant and thus were not retained in the final model. There was no group effect on the secondary driving outcomes: self-reported number of accidents or citations, *p*s ≥ 0.13. The two groups did not differ in accuracy on the street name search task (*t*(45) = 0.66, *p* = 0.51), with the OSA group averaging 4.42 mistakes and the control group averaging 4.11 mistakes.

### 3.3. Aim 2: Potential Mediating Role of Inattention and Sleepiness

We do not have eye tracker data for 8 adolescents with OSA and 10 controls due to device malfunction or participants wearing their own prescription glasses. One parent of an adolescent with OSA did not complete their BASC. As shown in Table 3, there were no group differences in the frequency of long eye glances away from the roadway, *F*(1, 27) = 0.83, *p* = 0.37, or the mean duration of long glances, *F*(1,27) = 3.30, *p* = 0.08. The distraction condition had a significant effect on the frequency of long eye glances, *F*(1, 27) = 7.21, *p* = 0.01, and the mean duration of long glances, *F*(1, 27) = 16.40, *p* = 0.004. The group-by-condition interaction was trending towards significance for the frequency of long glances, *p* = 0.06. Exploratory follow-ups revealed no group difference in long glance frequency under the distraction condition (effect size = 0.26, *p* = 0.50), while the OSA group had more long glances than the control group under the no distraction condition (effect size = −0.79, *p* = 0.046). There was no significant interaction between the group and distraction condition for the duration of long glances (*p* = 0.71). There were no significant group differences for the measures of attention or sleepiness in daily life. Given the lack of clear OSA-related differences on driving safety outcomes and putative mediators, we did not pursue additional mediation analyses.

## 4. Discussion

Motor vehicle crashes are the leading cause of death among teens [1]. It is clear that obstructive sleep apnea (OSA) affects the driving skills of adults, but research has focused on people who are middle-aged or older [11]. Because adolescents are new to the complex task of driving and behave differently than more experienced drivers [27,28,34], we need to better understand how OSA might affect them in particular. This study found that a distraction task negatively impacted adolescent drivers as expected. However, contrary to our hypotheses, those with OSA did not fare worse. There were no statistically significant group differences in the ability to maintain lane positioning, the frequency of long glances away from the road, or the average length of these glances. The group-by-distraction interactions were also not significant. If anything, an interaction effect that approached significance (*p* = 0.06) suggested greater resilience to distraction in terms of simulated vehicle control among the OSA group. 

Among our sample, adolescents with and without OSA also had similar levels of sleepiness and inattention. That contrasts with a prior study utilizing parent and teacher reports that found that adolescents with OSA had more attention problems than those without any sleep-disordered breathing [20]. Notably, that sample included many more youth with moderate/severe OSA (*n* = 42) than were in our current study (*n* = 10). The prior study also included younger participants and a wider age range (M_age_ = 13 years; range = 10–17) than the current study (M_age_ = 17 years; range = 16–19). 

We suspect that sampling issues loomed large in the current findings. Our OSA group had a median obstructive AHI of 4.40, with most (52%) having mild OSA (obstructive AHI < 5). This makes sense given our focus on community-based recruitment, rather than clinically referred youth; mild sleep-disordered breathing is far more common than more severe OSA in the general population [62,63]. Our prior work suggests that, when examining certain behavioral challenges (e.g., parent-reported attention problems), the effects were more evident for moderate/severe OSA (obstructive AHI > 5) than for mild OSA [20]. The findings from that study were subject to potential reporter biases, suggesting a need for complementary data from multiple reporters and/or from objective sources. Given the convergence of findings from both reporter-based and objective outcomes, the present findings are reassuring for the large majority of youth with mild OSA. 

However, it remains possible that those with more severe OSA might have shown increased impairment on the driving tasks. This may be particularly true for those with both significant breathing obstruction and substantial waking impairment, such as inattention or daytime sleepiness. Among adults with OSA, most studies suggest that daytime sleepiness and attention deficits predict driving deficits far more strongly than do physiologic measures of OSA severity, such as the number of obstructive events per hour asleep [14]. However, two population studies found higher rates of motor vehicle crashes among adults with undiagnosed OSA, independent of self-assessed sleepiness [63]. It is important to note that the potential differences between those with mild versus moderate OSA are not yet clear. This is further complicated by the fact that patients with mild OSA are often excluded from OSA research. For example, a recent review noted that many studies examining attention deficits in adults with OSA only included patients with moderate or severe OSA and excluded those with mild OSA [64]. 

### 4.1. Limitations

While mild OSA is prevalent in the general population, our study’s predominant focus on mild OSA is a limitation. It is not clear how well the current findings generalize to patients referred for clinical sleep concerns or those with more severe OSA. It is also possible that youth with more severe OSA, or those with more severe daytime symptoms, may opt to delay getting their drivers’ license. If that is the case, the impact of OSA on novice drivers would not show up until later than our study age range. It is also important to consider the generalizability of our findings to the broader adolescent population. While outside the scope of this study, anxiety, depression, and other medical conditions could impact driving performance and may influence whether a teen becomes licensed. Our sample was primarily White (89.8%) with household incomes > $100K (63.2%), which may limit generalizability. Lastly, although our study avoided the selection bias that occurs when using an entirely clinical sample, our recruitment approach (e.g., regional hospital networks and social media advertising) was not a random selection, so it could further reduce generalizability. 

Although the use of a driving simulator allowed us to maximize the internal validity of the study and to ethically incorporate a distraction task, an important limitation is that our primary driving outcome was not from on-road driving. It is reassuring that secondary outcomes related to real-world driving (e.g., number of accidents) also showed no group effect, but those were based on self-report, which may not be as reliable as objective data. Future research should consider assessing behavioral factors (e.g., seatbelt use and compliance with traffic rules) and objective driving outcomes. Notably, it can be challenging to gather objective data from official records, which are particularly shielded for minors by privacy laws. It is possible that the simulator context was a novel enough environment to mitigate the impact of OSA; however, sleep-restricted adolescents and adults with more severe OSA clearly show deficits in driving simulators [24,45,46,47]. It is also important to note that this fixed-base simulator is unable to provide as much feedback (e.g., g-forces) as larger, dynamic-frame simulators. Further, it remains possible that the impact of OSA may differ on more dynamic and varied driving courses, though prior data suggest that such courses may be less sensitive to inadequate sleep [24].

Lastly, our samples were relatively small, which could limit statistical power. Even so, the adolescents with OSA had slightly (non-significantly) better outcomes in the driving simulator than controls, which argues against a simple attribution of effects to underpowered analyses. Still, we recommend that future research consider a larger, more diverse clinical sample with more severe OSA, possibly incorporating older participants or those who are considering licensure but who may not yet be licensed. 

### 4.2. Conclusions

This study did not find any differences in simulated driving skills between adolescents with OSA and those without OSA. However, adolescents had relatively mild OSA in our study. Therefore, more work is needed to explore whether these findings hold true among adolescents with more severe OSA and/or those who are clinically referred due to both nocturnal breathing concerns and daytime symptoms. If research supported a link between OSA and adolescent driving safety, it could fundamentally alter OSA care by reframing diagnosis and treatment in terms of concrete, immediate dangers, rather than abstract, remote risks.

## Figures and Tables

**Table 1:** Demographics and sleep (*n* = 49).

	OSA (*n* = 21)	Control (*n* = 28)	*p*-Value ^1^
**Age**, mean (SD)	17.68 (0.67)	17.67 (1.08)	0.98
**Gender**, *n* (%)			0.67
Male	14 (66.67)	17 (60.71)	
Female	7 (33.33)	11 (39.29)	
**Race**, *n* (%)			0.32
White	18 (85.71)	26 (92.86)	
Black or African American	1 (4.76)	1 (3.57)	
Biracial	2 (9.52)	0 (0)	
Unknown/Not Reported	0 (0)	1 (3.57)	
**Ethnicity**, *n* (%)			0.39
Hispanic/Latino	2 (9.52)	1 (3.57)	
Not Hispanic/Latino	19 (90.48)	27 (96.43)	
**Academic Grade**, *n* (%)			0.25
As	4 (19.05)	10 (35.71)	
As and Bs	15 (71.43)	12 (42.86)	
Bs	0 (0)	3 (10.71)	
Bs and Cs	1 (4.76)	2 (7.17)	
Cs and Ds	1 (4.76)	1 (3.57)	
**Driving Experience (days)**, mean (SD)	489.86 (304.56)	518.75 (332.04)	0.76
**BMI Category**, *n* (%)			0.02
Healthy	6 (28.57)	16 (57.14)	
Overweight	4 (19.05)	6 (21.43)	
Obesity	5 (23.81)	4 (14.29)	
Severe Obesity	6 (28.57)	2 (7.14)	
**Household Income**, *n* (%)			0.56
<$70K	7 (33.33)	7 (25.00)	
$70–125K	6 (28.57)	6 (21.43)	
>125K	8 (38.10)	15 (53.57)	
**PSG Data**			
**Obstructive Apnea–Hypopnea Index**, median (range)	4.40 (1.5–24.9)	0.45 (0–1.3)	<0.001
**Periodic Limb Movement Index**, median (range)	0 (0–23.1)	0 (0–73.5)	0.66
**Arousal Index**, median (range)	12.3 (3.5–21.5)	6.30 (2.7–12.4)	<0.001
**Sleep Efficiency after Sleep Onset**, mean (SD)	82.68 (10.31)	84.06 (11.56)	0.67
**Sleep Period Time**, mean (SD)	478.44 (33.26)	477.51 (32.46)	0.92
**Average NREM O2 Saturation**, mean (SD)	96.43 (1.33)	96.58 (1.01)	0.67
**Average REM O2 Saturation**, mean (SD)	96.72 (1.47)	97.04 (0.93)	0.40
**% Time in N1**, mean (SD)	5.13 (2.85)	3.61 (1.90)	0.03
**% Time in N2**, mean (SD)	55.28 (7.48)	55.95 (7.03)	0.75
**% Time in N3**, mean (SD)	17.11 (4.20)	21.20 (5.01)	0.004
**% Time in REM**, mean (SD)	20.18 (6.57)	19.25 (4.67)	0.56
**Self-reported Sleep Patterns**			
**Time in Bed (school nights; hours)**, mean (SD)	7.68 (1.37)	7.66 (1.04)	0.93
**Time in Bed (weekends; hours)**, mean (SD)	9.25 (1.55)	9.18 (0.96)	0.87
**Sleep Midpoint (school nights; hh:mm)**, mean (SD)	02:55 (00:56)	02:53 (00:40)	0.87
**Sleep Midpoint (weekends; hh:mm)**, mean (SD)	04:39 (00:55)	05:03 (00:52)	0.13

^1^ *p*-values are based on two-sample *t*-test (continuous variables), Chi-square test (discrete variables), or Mann–Whitney U test (non-normally distributed variables; e.g., BMI category, obstructive apnea–hypopnea index, periodic limb movement index, and arousal index).

**Table 2:** Simulator and self-reported driving by condition.

	Measures of Central Tendency ^1^		Effect Size ^2^	*p*-Value
	OSA	Control		
**SD of Lane Position in Driving Stimulator**				
Distraction	1.46	1.56	−0.31	0.30
No Distraction	1.23	1.22	−0.02	0.93
**Self-Reported Driving History**				
Any Accident	55.00%	32.14%	2.58	0.11
Any Citation	19.05%	14.29%	1.41	0.66

^1^ For driving simulator outcomes, least square means are based on linear mixed-effect models including condition × distraction interaction. Raw percentages of any accident/citation are displayed for self-reported driving history. ^2^ Effect sizes and *p*-values are based on comparing group difference with/without distraction using Cohen’s d for driving simulator outcomes or odds ratios for self-reported driving history.

**Table 3:** Inattention and sleepiness by condition.

	Measures of Central Tendency ^1^		Effect Size ^2^	*p*-Value
	OSA	Control		
**Number of Long Glances in Driving Simulator**				
Distraction	20.50	18.72	0.26	0.50
No Distraction	12.62	18.00	−0.79	0.046
**Mean Duration of Long Glances in Driving Simulator**				
Distraction	13.86	19.99	−0.55	0.34
No Distraction	6.20	10.83	−0.41	0.58
**Inattention**				
Parent BASC	21.55 (2.09)	21.86 (2.66)	−0.13	0.67
Parent BRF	3.67 (4.04)	3.32 (3.86)	0.09	0.76
Teen BRF	5.52 (3.28)	6.29 (3.60)	−0.21	0.45
**Sleepiness**				
Teen Epworth	8.29 (4.04)	8.04 (3.96)	0.06	0.83
Parent BRF	2.24 (2.41)	2.07 (2.28)	0.07	0.81
Teen BRF	5.10 (3.22)	5.51 (3.16)	−0.13	0.66

^1^ For each driving simulator outcome, least square means are based on linear mixed-effect models including condition × distraction interaction. M (SD) is presented for inattention and sleepiness. ^2^ Effect sizes and *p*-values are based on comparing group difference with/without distraction for driving simulator outcomes or two-sample *t*-tests (inattention/sleepiness).

## Data Availability

The data presented in this study are available on request from the corresponding author. The data are not publicly available due to patient privacy.
